# Impact of Multimorbidity and Polypharmacy on Clinical Outcomes of Elderly Chinese Patients with Atrial Fibrillation

**DOI:** 10.3390/jcm11051370

**Published:** 2022-03-02

**Authors:** Agnieszka Kotalczyk, Yutao Guo, Yutang Wang, Gregory Y. H. Lip, on behalf of the ChiOTEAF Registry Investigators

**Affiliations:** 1 Liverpool Centre for Cardiovascular Science, University of Liverpool and Liverpool Heart & Chest Hospital, Liverpool L14 3PE, UK; babinskagnieszka@gmail.com (A.K.); dor_guoyt@hotmail.com (Y.G.); 2 Silesian Centre for Heart Diseases, Department of Cardiology, Congenital Heart Diseases and Electrotherapy, Medical University of Silesia, 41-800 Zabrze, Poland; 3 Sixth Medical Centre, Department of Pulmonary Vessel and Thrombotic Disease, Chinese PLA General Hospital, Beijing 100142, China; 4 Second Medical Centre, Department of Cardiology, Chinese PLA General Hospital, Beijing 100853, China; wyt301@yeah.net; 5 Aalborg Thrombosis Research Unit, Department of Clinical Medicine, Aalborg University, 9000 Aalborg, Denmark

**Keywords:** atrial fibrillation, China, management, multimorbidity, polypharmacy

## Abstract

Background: The co-incidence of multiple morbidities and polypharmacy is common amongst patients with atrial fibrillation (AF); however, data on their impact on clinical outcomes are scarce in Asian cohorts. Objective: To evaluate the impact of multimorbidity and polypharmacy on clinical outcomes and AF management among elderly Chinese patients. Methods: The ChiOTEAF registry is a prospective, multicenter nationwide study conducted from October 2014 to December 2018. Endpoints of interest were the composite outcome of all-cause death/any thromboembolism (TE), all-cause death, cardiovascular death, TE events, major bleeding, as well as AF management. Results: The eligible cohort included 6341 individuals (mean age 74.7 ± 10.7; 39.1% female), of whom 4644 (73.2%) had multimorbidity (defined as two or more chronic diseases), and 2262 (35.7%) were treated with five or more medications. There were 2775 (43.8%) patients on anticoagulant (OAC) use. On multivariate analysis, (i) multimorbidity was associated with a higher odds ratio of the composite outcome (OR: 2.04; 95% CI: 1.49–2.79), all-cause death (OR: 1.82; 95% CI: 1.31–2.54), cardiovascular death (OR: 2.05; 95% CI: 1.13–3.69), any TE (OR: 2.69; 95% CI: 1.29–5.62), and major bleeding (OR: 2.61; 95% CI: 1.25–5.45); (ii) polypharmacy was associated with a lower odds ratio of all-cause death (OR: 0.78; 95% CI: 0.63–0.96). The use of OAC was safe and was associated with a lower odds ratio of the composite outcome and all-cause death in all subgroups of patients. Conclusions: Multimorbidity and polypharmacy were common among elderly AF Chinese patients. Multimorbidity was an independent predictor of adverse clinical outcomes. The use of OAC was safe and significantly improved survival amongst AF patients with multimorbidity and polypharmacy.

## 1. Introduction

Atrial fibrillation (AF) is the most common sustained arrhythmia, and its increasing prevalence is driven by population aging and being overburdened with comorbidities [1,2,3]. The co-incidence of multiple morbidities is common amongst AF patients [4], affecting approximately 70–80% of elderly AF patients [5,6,7].

Previous studies confirmed that multimorbidity is associated with worse clinical outcomes among AF patients [8]; the all-cause death rate is six-fold higher than in AF patients without concomitant health problems [9]. Moreover, the diagnosis of heart failure, chronic obstructive pulmonary disease, and osteoporosis were attributed to a higher risk of all-cause death in AF patients with multimorbidity [10]. However, data on multimorbidity and polypharmacy among Asian patients with AF are scarce, especially among elderly individuals.

This analysis evaluates the prognosis and impact of multimorbidity and polypharmacy on clinical outcomes and AF management among elderly Chinese patients included in a prospective nationwide registry.

## 2. Materials and Methods

The Optimal Thromboprophylaxis in Elderly Chinese Patients with Atrial Fibrillation (ChiOTEAF) registry is a prospective cohort study conducted between October 2014 and December 2018 in 44 sites from 20 Chinese provinces. The study protocol has been previously published [11]. Consecutive AF patients presenting to cardiologists, neurologists, or surgeons were enrolled. Data were gathered by local investigators at enrollment and follow-up visits and reported into an electronic form. Follow-up visits were performed at 6 and 12 months, and then annually for the following 2 years. The present analysis was focused on the primary endpoint at the 1 year follow-up of the initial cohort.

### 2.1. Ethics Statement

The registry was approved by the Central Medical Ethics Committee of Chinese PLA General Hospital, Beijing, China (approval no S2014-065-01) and local institutional review boards. Written informed consent was obtained from all individual participants included in the study.

### 2.2. Objectives

The principal objectives were as follows: (i) to describe the baseline characteristics of patients with multimorbidity and polypharmacy; (ii) to evaluate the impact of multimorbidity and polypharmacy on clinical outcomes, including the composite outcome of all-cause death/any thromboembolism (TE; ischemic stroke, transient ischemic attack, or peripheral embolism), as well as individual endpoints of all-cause death, cardiovascular death, TE events, and major bleeding; (iii) to identify potential predictors of the composite outcome in patients with multimorbidity; (iv) to assess the impact of multimorbidity and polypharmacy on AF management; and (v) to assess the efficacy and safety of oral anticoagulation (OAC) among these subgroups.

### 2.3. Definitions

‘Multimorbidity’ was defined as the co-incidence of two or more morbidities (in addition to AF) at enrolment among patients with AF [12,13]. For our analysis, we included diabetes mellitus, lipid disorder, prior ischemic stroke, chronic kidney disease, heart failure, coronary artery disease, hypertension, chronic liver disease, chronic obstructive pulmonary disease, and sleep apnea.

‘Polypharmacy’ was defined as the concomitant use of five or more medications (regardless of the reasons and utility) at enrolment [14]. For our analysis, we included angiotensin-converting enzyme inhibitors, angiotensin II receptor blockers, β-blockers, statins, digoxin, amiodarone, propafenone, diuretics, calcium channel blockers, nitrates, insulin, sulfonylureas, biguanides, antiplatelet agents, and OACs.

The CHA_2_DS_2_-VASc score [15] and the HAS-BLED bleeding score [16] were used to assess the thromboembolic (TE) and bleeding risks. Bleeding events were categorized according to the ISTH definition [17]. AF management was described by OAC-use (including 12-month persistence) and the use of rate or rhythm control procedures (i.e., electrical or pharmacological cardioversion, AF ablation, and cardiac implantable electronic device implantation). The effectiveness of OACs was assessed by the odds of the composite outcome, all-cause death, cardiovascular death, TE events, and safety by the odds of major bleeding at 1-year follow-up amongst study subgroups. Other variables included in the registry and their definitions were designed to match the EORP-AF Long-term General Registry [18] (see Supplementary Data, Methods).

### 2.4. Study Outcomes

Thromboembolism included ischemic stroke, transient ischemic attack (TIA), pulmonary embolism, deep vein thromboembolism, and other thromboembolisms (peripheral embolism, atrial thrombus, and left atrial appendage thrombus, etc.).

Major bleeding was defined as clinically overt bleeding accompanied by one or more of the following: a decrease in the blood hemoglobin level of more than 2.0 g/dL or more over 24 h, the need for a transfusion of 2 or more units of packed red cells, the need for corrective surgery, or the bleeding at a critical site (extracranial, intraspinal, intraocular, pericardial, intraarticular, intramuscular with compartment syndrome, or retroperitoneal).

All-cause death included cardiac death, vascular death, and non-cardiovascular death. Cardiac death included death caused by STEMI/NSTEMI, heart failure (HF), arrhythmia, cardiac perforation/tamponade, and other deaths of cardiac origin. Vascular death included death ascribed to ischemic stroke, hemorrhagic stroke, systemic bleeding, peripheral embolism, and pulmonary embolism.

### 2.5. Statistical Analysis

Continuous variables were reported as mean ± standard deviation (SD); between-group comparisons were made using the Student’s *t*-test or the Mann–Whitney U test (based on distribution). Categorical variables were reported as counts and percentages; between-group comparisons were made by χ^2^ test. Patient-reported quality of life was assessed by the EuroQol five dimensions questionnaire (EQ-5D-5L) [19]. The quality of life was assessed based on the EQ summary index (ranged from 0 to 1; the score of 1 indicates the best health state) estimated from the EQ-5D-5L value set for China [20]. A logistic univariate regression analysis was used to assess the predictors of the composite outcome in the multimorbidity group. The significant variables of relevant clinical interests were subsequently included in a multivariate regression model. Finally, logistic regression analysis assessed the age-adjusted association between the (i) multimorbidity, (ii) polypharmacy and clinical outcomes, as well as AF management. We provided additional analyses for AF patients multimorbidity and polypharmacy (combined); as well as for the not-anticoagulated subgroup.

In all analyses, a *p* value < 0.05 was considered statistically significant. Statistical analysis was performed using SPSS^®^ version 24 (IBM Corp, Armonk, NY, USA).

## 3. Results

**Figure 1:**
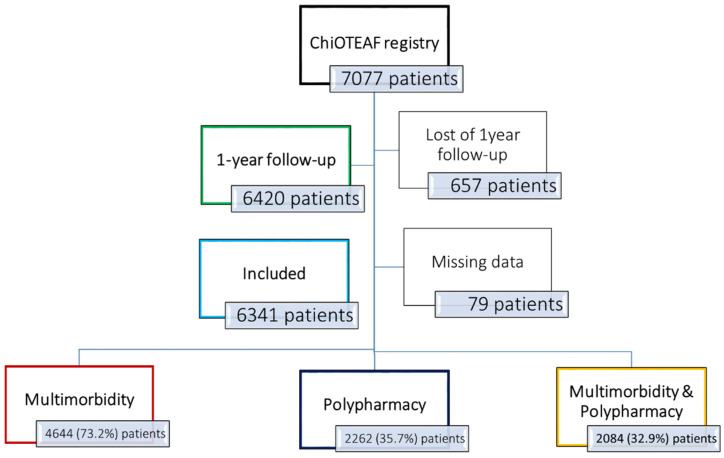
Flowchart of patient inclusion. ChiOTEAF: Optimal Thromboprophylaxis in Elderly Chinese Patients with Atrial Fibrillation.

The ChiOTEAF registry enrolled 7077 patients, of whom 657 (9.3%) were lost to follow-up at 1 year (Figure 1). The eligible cohort for this analysis included 6341 individuals (mean age 74.8 ± 10.7; 39.1% female); of these, 4644 (73.2%) had multimorbidity at baseline (the multimorbidity group; Supplementary Data Figure S1), and 2262 (35.7%) were treated with five or more medications (the polypharmacy group). A subgroup of 2084 (32.9%) patients had both multimorbidity and polypharmacy. Baseline characteristics are reported in Table 1.

Patients with multimorbidity were older (mean age of 76.5 ± 10.2 vs. 69.9 ± 10.4; *p* < 0.001), with a higher risk of stroke (mean CHA_2_DS_2_VASc score of 4.1 ± 1.6 vs. 2.3 ± 1.1; *p* < 0.001) and bleeding (HAS-BLED score of 2.4 ± 1.1 vs. 1.4 ± 0.8; *p* < 0.001). In the multimorbidity group, the most prevalent comorbidities were hypertension (75.8%), coronary artery disease (62.6%), and lipid disorders (57.2%).

Patients with polypharmacy were older (mean age of 76.4 ± 10.0 vs. 73.8 ± 10.9; *p* < 0.001), with a higher proportion of OAC-treated patients (49.8% vs. 40.4%; *p* < 0.001) compared with the non-polypharmacy group. Interestingly, a higher persistence to OAC therapy at 12-month was observed in the polypharmacy group (44.8% vs. 34.6%; *p* < 0.001). The most prevalent medications in the polypharmacy group were statins (83.7%), β-blockers (76.6%), and antiplatelets (59.1%).

### 3.1. Mortality and Morbidity

Of the overall study cohort, 435 (6.8%) patients died between the enrolment and the 1-year follow-up visit; and 390 (89.6%) deaths occurred in the multimorbidity group; whilst 151 (34.7%) died in the polypharmacy group. Rates of adverse events at 1 year follow-up are reported in Supplementary Data Table S1.

Patients with multimorbidity had higher rates of the composite outcome (9.9% vs. 2.8%; *p* < 0.001), all-cause death (8.4% vs. 2.5%; *p* < 0.001), cardiovascular death (2.3% vs. 0.8%; *p* < 0.001), any TE (2.0% vs. 0.5%; *p* < 0.001), and major bleeding (2.0% vs. 0.5%; *p* < 0.001), compared to the non-multimorbidity group. Multimorbidity was associated with a higher odds ratio (OR) of composite outcome (OR: 2.04; 95% confidence interval [CI]: 1.49–2.79), all-cause death (OR: 1.82; 95% CI: 1.31–2.54), cardiovascular death (OR: 2.05; 95% CI: 1.13–3.69), any TE (OR: 2.69; 95% CI: 1.29–5.62), and major bleeding (OR: 2.61; 95% CI: 1.25–5.45)—Table 2.

In patients with polypharmacy (vs. non-polypharmacy group), no statistically significant differences were found in the rates of these clinical outcomes. However, polypharmacy was associated with a lower (age-adjusted) odds ratio of all-cause death (OR: 0.78; 95% CI: 0.63–0.96)—Table 2.

### 3.2. Multivariate Analysis

During multivariate analysis (Table 3), independent variables associated with the composite outcome of all-cause death/any TE in patients with multimorbidity were as follows: (i) the use of OAC (OR: 0.49; 95% CI: 0.38–0.63) and polypharmacy (OR: 0.73; 95% CI: 0.58–0.91) were protective; and (ii) age (OR: 1.11; 95% CI: 1.09–1.12), heart failure (OR: 2.14; 95% CI: 1.71–2.69), prior ischemic stroke (OR: 1.47; 95% CI: 1.19–1.82), chronic kidney disease (OR: 1.75; 95% CI: 1.38–2.21), and chronic obstructive pulmonary disease (OR: 1.61; 95% CI: 1.25–2.06) were associated with greater mortality.

### 3.3. AF Management

Multimorbidity was associated with a higher odds ratio of antiplatelet use (OR: 2.87; 95% CI: 2.51–3.27), OAC persistence at 12 months (OR: 1.43; 95% CI: 1.26–1.62), and cardiovascular implantable electronic device use (OR: 1.54; 95% CI: 1.19–1.99), but a lower proportion of AF ablation (7.8% vs. 23.2%; *p* < 0.001); see Supplementary Data Table S2. Patients with five or more comorbidities had the lowest odds ratio of OAC prescription (OR: 0.63; 95% CI: 0.51–0.78); see Supplementary Data Table S3.

Polypharmacy was associated with an OAC prescription (OR: 1.66; 95% CI: 1.49–1.85), OAC persistence at 12 months (OR: 1.59; 95% CI: 1.42–1.78), pharmacological cardioversion (OR: 1.92; 95% CI: 1.61–2.29), as well as antiplatelet use (OR: 3.05; 95% CI: 2.74–3.39), but a lower odds ratio of AF ablation (OR: 0.66; 95% CI: 0.55–0.79).

### 3.4. Impact of Oral Anticoagulation

In the overall cohort, OAC use was associated with a lower odds ratio of the composite outcome and all-cause death, regardless of the number of comorbidities (Supplementary Data Table S4). In patients with polypharmacy, the use of OAC was related to a lower odds ratio of the composite outcome (polypharmacy: OR: 0.32; 95% CI: 0.23–0.45) and all-cause death (OR: 0.33; 95% CI: 0.23–0.48); see Supplementary Data Table S5.

We performed a separate analysis of clinical outcomes among non-anticoagulated patients (Supplementary Data, Table S5). In this subgroup, multimorbidity was associated with a higher odds ratio of the composite outcome (OR: 1.89; 95% CI: 1.33–2.68), all-cause death (OR: 1.65; 95% CI: 1.15–2.38), and any TE (OR: 3.77; 95% CI: 1.36–10.45).

## 4. Discussion

The ChiOTEAF registry provides contemporary management among elderly Chinese AF patients, along with 1 year follow-up data [21]. This analysis focused on multimorbidity and polypharmacy, evaluating their impact on clinical outcomes and AF management. To our knowledge, this is the first large-cohort study assessing these issues in Asian patients.

The primary findings of the present study are as follows: (i) 73.2% of elderly AF patients had multimorbidity, while polypharmacy was present in 35.7% of patients; (ii) multimorbidity was associated with higher rates of the composite outcome, all-cause death, cardiovascular death, any TE, and major bleeding; and was an independent predictor of the adverse clinical outcomes; (iii) the independent predictors of the composite outcome among multimorbidity patients were the non-use of OAC, age, heart failure, prior ischemic stroke, chronic kidney disease, chronic obstructive pulmonary disease, and non-polypharmacy; (iv) multimorbidity was associated with a higher odds ratio of antiplatelet use and rate control strategy; and (v) OAC use was safe and improved survival in AF patients with multimorbidity, as well as polypharmacy.

Multimorbidity and polypharmacy are commonly associated with older patients and impairment of their clinical status [5,22,23]. It is challenging for clinicians to prescribe OACs to elderly patients with comorbidities and increased polypharmacy because of unclear clinical net benefit. The present study shows how OAC has reduced the composite outcome of all-cause death and any thromboembolism in this complex AF population with multimorbidity, re-emphasizing the importance of optimal thromboprophylaxis. Given that our study cohort was comprised of elderly AF patients, the vast majority (73.2%) had multimorbidity, and a third had polypharmacy. Previous studies have confirmed that various risk factors and comorbidities concomitant with AF (such as older age, heart failure, coronary artery disease, and diabetes) are associated with the risk of hospitalization and death [24]. In our analysis, the independent predictors of the composite outcome (all-cause death/any TE) among multimorbidity patients were the non-use of OAC, age, heart failure, prior ischemic stroke, chronic kidney disease, chronic obstructive pulmonary disease, and non-polypharmacy. Furthermore, we found that multimorbidity (but not polypharmacy) was associated with higher rates of the composite outcome, all-cause death, cardiovascular death, any TE, and major bleeding, and was an independent predictor of the adverse clinical outcomes.

Instead, polypharmacy was protective against the composite outcome of all-cause death/any TE. These may reflect the adequate care provided to these complex patients, including a higher number of patients persistent to OAC at 12 months compared with the non-polypharmacy group. Our previous analysis showed a slightly better persistence among patients prescribed with a NOAC (than warfarin); in particular among those treated with rivaroxaban (the once-daily dosing regimen), compared with dabigatran (twice-daily regimen) [25].

Furthermore, the differences in AF management were evident across the study cohort. Of note, the rhythm control strategy was limited, and AF ablation was performed only in 11.9% of the overall study cohort. Consistent with previous data [26], patients with multimorbidity were more often treated with digoxin; while amiodarone and propafenone were used in non-multimorbidity patients. Multimorbidity and polypharmacy were associated with a lower odds ratio of AF ablation.

Another aspect is the low number of anticoagulated patients (42% of patients in the multimorbidity group), despite a high risk of TE events. In our previous study, guideline adherent OAC use was found to be safe among elderly Chinese AF patients [25]. Notably, in this analysis, the efficacy and safety of OACs were sustained regardless of the number of comorbidities or the presence of polypharmacy. Interestingly, we found that patients with multimorbidity were less likely to be prescribed with a NOAC; instead, these patients had a three-fold higher odds ratio of antiplatelet use. This may reflect the ‘real-life’ clinical practice, in which elderly with multimorbidity or polypharmacy are not prescribed with OAC due to a fear of bleeding and drug–drug interactions [27]. Of note, among non-anticoagulated patients, multimorbidity was significantly attributed to the composite outcome, as well as all-cause death and TE events.

Previous studies showed that NOACs were more effective than warfarin, and (at least) just as safe in patients with polypharmacy [28,29]. In the recent analysis, efficacy and safety of apixaban were preserved in AF patients with multimorbidity, extending trial results to the most complex individuals [30]. Of note, the ARISTOPHANES subgroup analysis evaluated the effectiveness and safety of OAC in AF patients with multimorbidity (defined as six or more comorbidities) [31]. Apixaban and rivaroxaban were associated with a lower risk of TE, while apixaban and dabigatran had a lower risk of major bleeding than warfarin [31].

Multimorbidity and polypharmacy are highly prevalent in AF patients, but it should not be a reason to withhold OAC therapy. Instead, these patients should be involved into an integrated holistic care model to improve their clinical outcomes and quality of life [32,33]. Due to fewer drug–drug interactions (compared with VKA), NOACs should be preferred in AF patients with polypharmacy and multimorbidity [34].

### Limitations

The primary limitation of the ChiOTEAF registry is its observational character. Patients were enrolled in 44 centers for a relatively long recruitment period, implying a potential bias and variability in the local AF management. We found that a moderate proportion of patients were lost to follow-up (9.3%), and the causes of 67 deaths (15.4%) were unknown. The number of adverse clinical events, as well as AF-related procedures, may be underreported. Data on anticoagulation control, the use of traditional Chinese medicines, and management of comorbidities/cardiovascular risk factors were not available and could not be considered in the analysis.

## 5. Conclusions

Multimorbidity and polypharmacy were common among elderly AF Chinese patients. Multimorbidity was an independent predictor of adverse clinical outcomes. The use of OAC was safe and significantly improved survival amongst AF patients with multimorbidity and polypharmacy.

## Figures and Tables

**Table 1:** Baseline characteristics of the study cohort.

	Total N = 6341 *n* (%)	Multi Morbidity Group N = 4644 *n* (%)	Non-Multi Morbidity Group N = 1697 *n* (%)	*p*	Polypharmacy Group N = 2262 *n* (%)	Non-Polypharmacy Group N = 4079 *n* (%)	*p*
Age *; years	74.7 ±10.7	76.5 ±10.2	69.9 ±10.4	<0.001	76.4 ±10.0	73.8 ±10.9	<0.001
Female gender	2477 (39.1)	1811 (39.0)	666 (39.2)	0.857	929 (41.1)	1548 (38.0)	0.015
BMI * [kg/m^2^]	24.1 ±3.6	24.2 ±3.7	23.9 ±3.5	0.006	24.6 ±3.6	23.9 ±3.6	<0.001
First diagnosed AF	941 (17.2)	675 (17.2)	266 (17.4)	0.886	346 (18.3)	595 (16.7)	0.120
Medical history							
Diabetes mellitus	1611 (26.2)	1596 (34.4)	65 (38.8)	<0.001	939 (41.5)	722 (17.7)	<0.001
Hypertension	4045 (63.8)	3518 (75.8)	527 (31.1)	<0.001	1789 (79.1)	2256 (55.3)	<0.001
Heart failure	2258 (35.6)	2109 (45.4)	149 (8.8)	<0.001	1144 (50.6)	1144 (27.3)	<0.001
Coronary artery disease	3006 (47.4)	2909 (62.6)	97 (5.7)	<0.001	1513 (66.9)	1493 (36.6)	<0.001
Liver disease	253 (4.0)	230 (5.0)	23 (1.4)	<0.001	86 (3.8)	167 (4.1)	0.569
Lipid disorder	2788 (44.0)	2655 (57.2)	133 (4.8)	<0.001	1354 (59.9)	1434 (35.2)	<0.001
Prior ischemic Stroke	1580 (24.9)	1517 (32.7)	63 (3.7)	<0.001	700 (44.3)	880 (21.6)	<0.001
Chronic kidney disease	784 (12.4)	770 (16.6)	14 (1.8)	<0.001	372 (16.4)	412 (10.1)	<0.001
COPD	595 (9.4)	576 (12.4)	19 (1.1)	<0.001	247 (10.9)	348 (8.5)	0.002
Sleep apnea	205 (3.2)	197 (4.2)	8 (0.5)	<0.001	99 (4.4)	106 (2.6)	<0.001
Dementia	221 (3.5)	198 (4.3)	23 (1.4)	<0.001	90 (4.0)	131 (3.2)	0.111
Cancer	684 (10.8)	494 (10.6)	190 (11.2)	0.525	189 (8.4)	495 (12.1)	<0.001
Hyperthyroidism (*n* = 6198)	109 (1.8)	74 (1.6)	35 (2.1)	0.220	31 (1.4)	78 (2.0)	0.116
Hypothyroidism (*n* = 6194)	249 (4.0)	212 (4.7)	37 (2.2)	<0.001	111 (5.0)	138 (3.5)	0.003
Prior major bleeding (*n* = 6338)	265 (4.2)	235 (5.1)	30 (1.8)	<0.001	90 (4.0)	175 (4.3)	0.552
CHA_2_DS_2_VASc * (*n* = 5908)	3.6 ±1.7	4.1 ±1.6	2.3 ±1.1	<0.001	4.3 ±1.7	3.2 ±1.6	<0.001
HAS-BLED * (*n* = 6013)	2.2 ±1.1	2.4 ±1.1	1.4 ±0.8	<0.001	2.5 ±1.1	1.9 ±1.1	<0.001
Medications							
OAC	2775 (43.8)	1947 (41.9)	828 (48.8)	<0.001	1127 (49.8)	1648 (40.4)	<0.001
- VKA	1329 (21.0)	960 (20.7)	369 (21.7)	0.353	569 (25.2)	760 (18.6)	<0.001
- NOAC	1446 (22.8)	987 (21.3)	459 (27.0)	<0.001	557 (24.6)	889 (21.8)	0.010
Antiplatelet	2604 (41.1)	2232 (48.1)	372 (14.3)	<0.001	1337 (59.1)	1267 (31.1)	<0.001
- Aspirin (*n* = 6340)	1803 (28.4)	1538 (33.1)	265 (15.6)	<0.001	938 (41.5)	865 (21.2)	<0.001
- Clopidogrel (*n* = 6337)	1259 (19.9)	1133 (24.4)	126 (7.4)	<0.001	690 (30.5)	569 (14.0)	<0.001
- Ticagrelor (*n* = 6340)	25 (0.4)	19 (0.4)	6 (0.4)	0.754	14 (0.6)	11 (0.3)	0.033
Dual Antiplatelet (*n* = 6336)	540 (8.5)	496 (10.7)	44 (8.1)	<0.001	326 (14.4)	214 (5.2)	<0.001
OAC + Antiplatelet	392 (6.2)	351 (7.6)	41 (2.4)	<0.001	311 (13.7)	81 (2.0)	<0.001
Digoxin	745 (11.7)	604 (13.0)	141 (8.3)	<0.001	487 (21.5)	258 (6.3)	<0.001
β-blockers	3360 (53.0)	2710 (58.4)	650 (38.3)	<0.001	1732 (76.6)	1628 (39.9)	<0.001
Amiodarone	912 (14.4)	577 (12.4)	335 (19.7)	<0.001	398 (17.6)	514 (12.6)	<0.001
Propafenone	288 (4.5)	158 (3.4)	130 (7.7)	<0.001	82 (3.6)	206 (5.1)	0.009
ACE-I	835 (13.2)	717 (15.4)	118 (7.0)	<0.001	509 (22.5)	326 (8.0)	<0.001
ARB	1633 (25.8)	1357 (29.2)	276 (16.3)	<0.001	968 (42.8)	665 (16.3)	<0.001
Calcium channel blockers	1703 (26.9)	1444 (31.1)	259 (15.3)	<0.001	971 (42.9)	732 (17.9)	<0.001
Diuretics	1794 (28.3)	1563 (33.7)	231 (13.6)	<0.001	1134 (50.1)	660 (16.2)	<0.001
Statins	3583 (56.5)	3123 (67.2)	460 (27.1)	<0.001	1893 (83.7)	1690 (41.4)	<0.001
Insuline	450 (7.1)	430 (9.3)	20 (1.2)	<0.001	333 (14.7)	117 (2.9)	<0.001
Sulfynylureas	325 (5.1)	308 (6.6)	17 (1.0)	<0.001	242 (10.7)	83 (2.0)	<0.001
Biguanide	400 (6.3)	374 (8.1)	26 (1.5)	<0.001	293 (13.0)	107 (2.6)	<0.001
Nitrates	1549 (24.4)	1417 (30.5)	132 (7.8)	<0.001	1083 (47.9)	466 (11.4)	<0.001
Polypharmacy	2262 (35.7)	2084 (44.9)	178 (10.5)	<0.001	-	-	-
Quality of life							
EHRA score * (*n* = 3888)	1.6 ±0.5	1.6 ±0.5	1.5 ±0.5	<0.001	1.6 ±0.5	1.5 ±0.5	0.047
EQ index * (*n* = 5701)	0.83 ±0.18	0.81 ±0.19	0.88 ±1.48	<0.001	0.80 ±0.19	0.84 ±0.17	<0.001
AF management							
OAC persistence at 12-month (*n* = 5931)	2267 (38.2)	1713 (40.0)	554 (33.5)	<0.001	951 (44.8)	1316 (34.6)	<0.001
Electrical cardioversion (*n* = 6336)	43 (0.7)	28 (0.6)	15 (0.9)	0.227	17 (0.8)	26 (0.6)	0.597
Pharmacological cardioversion (*n* = 6336)	571 (9.0)	434 (9.4)	137 (8.1)	0.118	289 (12.8)	282 (6.9)	<0.001
AF ablation (*n* = 6336)	754 (11.9)	360 (7.8)	394 (23.2)	<0.001	182 (8.0)	572 (14.0)	<0.001
CIED (*n* = 6336)	511 (8.1)	432 (9.3)	79 (4.7)	<0.001	209 (9.2)	302 (7.4)	0.010

* Mean ± standard deviation. ACE-I—angiotensin-converting enzyme inhibitor; AF—atrial fibrillation; ARB—angiotensin II receptor blocker; BMI—body mass index; CHA_2_DS_2_VASc—Congestive heart failure/left ventricular dysfunction, Hypertension, Age ≥ 75 (doubled), Diabetes, Stroke (doubled), Vascular disease, Age 65–74, female Sex; COPD—chronic obstructive pulmonary disease; CIED—cardiac implantable electronic device; EHRA—European Heart Rhythm Association; EQ—EuroQoL; HAS-BLED—hypertension, abnormal renal/liver function, stroke, bleeding history or predisposition, the labile international normalized ratio [INR], elderly, and drugs/alcohol use; NOAC—non-vitamin K antagonist oral anticoagulant; OAC—oral anticoagulation; VKA—vitamin K antagonist.

**Table 2:** The effects of multimorbidity and polypharmacy on clinical outcomes (composite outcome; all-cause death; cardiovascular death; any thromboembolism; major bleeding).

	Multimorbidity		Polypharmacy		Multimorbidity and Polypharmacy	
	Odds Ratio *	95% CI	Odds Ratio *	95% CI	Odds Ratio *	95% CI
Composite outcome ^#^	2.04	1.49–2.79	0.83	0.68–1.01	0.87	0.71–1.06
All-cause death	1.82	1.31–2.54	0.78	0.63–0.96	0.81	0.65–1.01
Cardiovascular death	2.05	1.13–3.69	1.02	0.71–1.48	1.08	0.74–1.56
Any TE	2.69	1.29–5.62	1.03	0.69–1.54	1.12	0.75–1.69
Major bleeding	2.61	1.25–5.45	1.16	0.78–1.74	1.21	0.81–1.82

* Adjusted for age. ^#^ Composite outcome of all-cause death/any thromboembolism. TE—thromboembolism; CI—confidence interval.

**Table 3:** Predictors of the composite outcome (all-cause death/any thromboembolism) among patients with atrial fibrillation and multimorbidity.

	Univariate			Multivariate		
	Odds Ratio	95% CI	*p*	Odds Ratio	95% CI	*p*
OAC	0.33	0.27–0.42	<0.001	0.49	0.38–0.63	<0.001
Age	1.14	1.12–1.15	<0.001	1.11	1.09–1.12	<0.001
Female gender	0.78	0.63–0.95	0.014	-	-	-
Diabetes mellitus	1.06	0.86–1.29	0.601			
Hypertension	0.78	0.63–0.97	0.025	-	-	-
Heart failure	2.88	2.34–3.53	<0.001	2.14	1.71–2.69	<0.001
Coronary artery disease	1.31	1.07–1.61	0.010	-	-	-
Prior ischemic stroke	1.85	1.52–2.25	<0.001	1.47	1.19–1.82	<0.001
Chronic kidney disease	2.84	2.30–3.51	<0.001	1.75	1.38–2.21	<0.001
COPD	3.32	2.65–4.15	<0.001	1.61	1.25–2.06	<0.001
Sleep apnea	0.99	0.62–1.59	0.964			
Antiplatelet therapy	1.08	0.89–1.31	0.407			
Polypharmacy	0.77	0.63–0.93	0.008	0.73	0.58–0.91	0.005

CI—confidence interval; COPD—chronic obstructive pulmonary disease; OAC—oral anticoagulation.

## Data Availability

The datasets used and analyzed during the current study are available from the corresponding author on reasonable request.

## Supplementary Materials

The following supporting information can be downloaded at: https://www.mdpi.com/article/10.3390/jcm11051370/s1, Methods: Definitions; Table S1: Rates of the clinical outcomes: composite outcome; all-cause death; cardiovascular death; any thromboembolism; and major bleeding among patients with multimorbidity (A), polypharmacy (B), and multimorbidity and polypharmacy (C); Table S2: The effects of multimorbidity and polypharmacy on the AF management; Table S3: The number of comorbidities and their effect on OAC prescription among AF patients; Table S4: Efficacy and safety of OAC in AF patients regarding the number of comorbidities, in AF patients with polypharmacy, and in AF patients with multimorbidity and polypharmacy; Table S5: The effects of multimorbidity and polypharmacy on clinical outcomes (composite outcome; all-cause death; cardiovascular death; any thromboembolism; major bleeding) among non-anticoagulated patients; Figure S1: Proportion of patients with atrial fibrillation according to number of comorbidities; List of ChiOTEAF registry investigators.
