# Supplementary material for: Impact of Multimorbidity and Polypharmacy on Clinical Outcomes of Elderly Chinese Patients with Atrial Fibrillation
Source: J Clin Med. 2022 Mar 2;11(5):1370. doi: 10.3390/jcm11051370 (PMC8910929; doi:10.3390/jcm11051370)
Supplement: Supplementary file 1 [file jcm-11-01370-s001.zip › jcm-1578356-supplementary.pdf]

DATA SUPPLEMENT

CONTENTS

- METHODS: Definitions

- **Table S1.** Rates of the clinical outcomes: composite outcome; all-cause death; cardiovascular death; any thromboembolism; major bleeding among patients with multimorbidity (A), polypharmacy (B), multimorbidity and polypharmacy (C).

- Table S2. The effects of multimorbidity and polypharmacy on the AF management.

- **Table S3.** The number of comorbidities and their effect on OAC prescription among AF patients.

- **Table S4**. Efficacy and safety of OAC in AF patients regarding the number of comorbidities; in AF patients with polypharmacy; and in AF patients with multimorbidity and polypharmacy.

- Table S5. The effects of multimorbidity and polypharmacy on clinical outcomes (composite outcome; all-cause death; cardiovascular death; any thromboembolism; major bleeding) among non-anticoagulated patients.

- **Figure S1.** Proportion of patients with atrial fibrillation according to number of comorbidities.

- List of ChiOTEAF Registry Investigators

METHODS Definitions:

First diagnosed Atrial fibrillation (AF): Every patient who presents with AF for the first time is considered a patient with first diagnosed AF, irrespective of the duration of the arrhythmia or the presence and severity of AF-related symptoms.

Diabetes mellitus: HbA1C 6.5% or fasting plasma glucose 126 mg/dl (7.0 mmol/l). Fasting is defined as no caloric intake for at least 8 h.* OR 2-h plasma glucose 200 mg/dl (11.1mmol/l) during an oral glucose tolerance test (OGTT) OR In a patient with classic symptoms of hyperglycemia OR hyperglycemic crisis, a random plasma glucose 200 mg/dl (11.1 mmol/l) OR Treatment with oral hypoglycemic agent and/or insulin.

Hypertension: Indicate if the patient has a history of hypertension diagnosed and/or treated before the current hospitalization.

Presence of resting blood pressure greater than 140 mmHg systolic or 90 mmHg diastolic on at least 2 occasions or current anti-hypertensive pharmacological treatment.

Chronic kidney disease: Chronic kidney disease is defined as kidney damage (structural abnormality or persistent hematuria and/or proteinuria) or glomerular filtration rate <60 mL/min/1.73 m2 for 3 months or more, irrespective of cause.

Chronic Obstructive Pulmonary Disease: Lung disease characterized by chronic obstruction of lung airflow that interferes with normal breathing and is not fully reversible.

Chronic liver disease: patients presenting with signs and symptoms of chronic liver disease or has risk factors with confirmed laboratory abnormalities or positive screen for serologic markers of liver disease.

Heart failure: Symptoms (e.g. breathlessness, ankle swelling, fatigue) and signs (elevated jugular venous pressure, pulmonary crackles, and displaced apex beat) resulting from an abnormality of cardiac structure or function.

Coronary artery disease: Indicate if the patient has a history of stable angina, unstable angina or myocardial infarction. Acute coronary syndrome: i) acute coronary syndrome with ST segment elevation - acute chest pain with persistent (>20 min) ST-segment elevation or (presumably) new left bundle branch block, ii) acute coronary syndrome without persistent ST segment elevation - acute chest pain with persistent or transient ST-segment depression or T-

wave inversion, flat T waves, pseudo-normalization of T waves, or no ECG changes; if rise/fall in cardiac troponins was registered, acute coronary syndrome without persistent ST segment elevation was designated as non-ST elevation myocardial infarction, while normal troponin levels denoted unstable angina pectoris. Stable coronary artery disease: episodes of reversible myocardial demand/supply mismatch, related to ischemia or hypoxia, which are usually inducible by exercise, emotion or other stress and are reproducible but, which may also be occurring spontaneously.

Ischemic stroke: A focal neurologic deficit, from a nontraumatic cause, lasting at least 24 hours.

Transient ischemic stroke: A focal neurologic deficit, from a nontraumatic cause, lasting less than 24 hours

Peripheral embolism: clinical history consistent with an acute loss of blood flow to a peripheral artery (or arteries) supported by evidence of embolism.

Sleep apnea: Repetitive collapse of the upper airway during sleep with chronic intermittent hypoxia and recurrent arousals

Dementia: Indicate if the patient has a history of cognitive impairment (greater than for normal aging). Dementia is defined as a syndrome characterized by impairment of multiple higher cortical functions, including memory, thinking, orientation, calculation, learning capacity, language and judgement due to disease of the brain.

Lipid disorder: Indicate if the patient has a history of any lipid disorder and/or treated before the current hospitalization.

Intracranial bleeding: Indicate if the patient has a history of epidural, subdural, subarachnoid, or intracerebral hemorrhage.

Extracranial bleeding: Indicate if the patient has a history of gastrointestinal, respiratory, urogenital, mouth, skin/soft tissue/muscle, joints, or spleen bleeding. Bleeding events were categorized according with the International Society on Thrombosis and Haemostasis (ISTH) definition.

Table S1. Rates of the clinical outcomes: composite outcome; all-cause death; cardiovascular death; any thromboembolism; major bleeding among patients with multimorbidity (A), polypharmacy (B), multimorbidity and polypharmacy (C).

**A**	**Multimorbidity N=4644** n (%)	**Non-multimorbidity N=1697** n (%)	**P**
**Composite outcome#**	461 (9.9)	48 (2.8)	<0.001
**All-cause death**	390 (8.4)	43 (2.5)	<0.001
**Cardiovascular death**	109 (2.3)	13 (0.8)	<0.001
**Any TE**	92 (2.0)	8 (0.5)	<0.001
**Major bleeding**	90 (2.0)	8 (0.5)	<0.001
**B**	**Polypharmacy N=2262** n (%)	**Non-polypharmacy N=4079** n (%)	**P**
**Composite outcome#**	184 (8.1)	325 (8.0)	0.815
**All-cause death**	151 (6.7)	282 (6.9)	0.719
**Cardiovascular death**	48 (2.1)	74 (1.8)	0.393
**Any TE**	40 (1.8)	60 (1.5)	0.369
**Major bleeding**	42 (1.9)	56 (1.4)	0.138
**C**	**Multimorbidity and polypharmacy N=2084** n (%)	**Non-multimorbidity and polypharmacy N=4257**	**P**

	n (%)		
**Composite outcome#**	180 (8.6)	329 (7.7)	0.211
**All-cause death**	147 (7.1)	286 (6.7)	0.619
**Cardiovascular death**	47 (2.3)	75 (1.8)	0.179
**Any TE**	40 (1.9)	60 (1.4)	0.128
**Major bleeding**	41 (2.0)	57 (1.3)	0.058

Composite outcome of all-cause death/any thromboembolism

TE – thromboembolism; CI – confidence interval

Table S2. The effects of multimorbidity and polypharmacy on the AF management.

	**Multimorbidity**		**Polypharmacy**		**Multimorbidity and polypharmacy**	
	Odds ratio*	95% CI	Odds ratio*	95% CI	Odds ratio*	95% CI
OAC prescription	0.96	0.85-1.08	**1.66**	**1.49-1.85**	**1.49**	**1.33-1.66**
Antiplatelet	**2.87**	**2.51-3.27**	**3.05**	**2.74-3.39**	**3.21**	**2.87-3.58**
OAC persistence at 12-month	**1.43**	**1.26-1.62**	**1.59**	**1.42-1.78**	**1.53**	**1.37-1.71**
AF ablation	**0.41**	**0.35-0.48**	**0.66**	**0.55-0.79**	**0.53**	**0.43-0.64**
CIED	**1.54**	**1.19-1.99**	1.14	0.94-1.37	1.16	0.96-1.39
Electrical cardioversion	0.91	0.47-1.74	1.36	0.73-2.53	1.30	0.69-2.47
Pharmacological cardioversion	1.08	0.87-1.33	**1.92**	**1.61-2.29**	**1.76**	**1.48-2.10**

*Adjusted for age.

AF – atrial fibrillation; CI – confidence interval; CIED - cardiac implantable electronic device; OAC – oral anticoagulants.

Data Supplement Table S3. The number of comorbidities and their effect on OAC prescription among AF patients.

**AF and comorbidites**	**OAC prescription**	
	Odds ratio	95% CI
0	ref	ref
1	1.11	0.91-1.36
2	0.98	0.81-1.19
3	0.82	0.68-0.99
4	0.78	0.64-0.96
≥5	0.63	0.51-0.78

AF – atrial fibrillation; CI – confidence interval; OAC – oral anticoagulants

Data Supplement Table S4. Efficacy and safety of OAC in AF patients regarding the number of comorbidities; in AF patients with polypharmacy; and in AF patients with multimorbidity and polypharmacy.

**AF and comorbidites**	**Composite outcome**		**All-cause death**		**Major bleeding**	
	Odds ratio	95% CI	Odds ratio	95% CI	Odds ratio	95% CI
0	0.16	0.04-0.69	0.08	0.01-0.59	-	-
1	0.23	0.09-0.55	0.21	0.08-0.56	-	-
2	0.26	0.15-0.46	0.21	0.11-0.42	0.84	0.33-2.09
3	0.58	0.39-0.87	0.53	0.34-0.84	1.37	0.56-3.31
4	0.28	0.16-0.47	0.26	0.14-0.46	0.56	0.22-1.45
≥5	0.29	0.19-0.45	0.34	0.22-0.53	0.88	0.41-1.89
**Polypharmacy**	0.32	0.23-0.45	0.33	0.23-0.48	1.03	0.55-1.85
**Multimorbidity and polypharmacy**	0.34	0.24-0.49	0.36	0.24-0.52	1.14	0.61-2.12

AF – atrial fibrillation; CI – confidence interval.

Data Supplement Table S5. The effects of multimorbidity and polypharmacy on clinical outcomes (composite outcome; all-cause death; cardiovascular death; any thromboembolism; major bleeding) among non-anticoagulated patients.

	**Multimorbidity**		**Polypharmacy**		**Multimorbidity and polypharmacy**	
	Odds ratio*	95% CI	Odds ratio*	95% CI	Odds ratio*	95% CI
**Composite outcome#**	1.89	1.33-2.68	0.89	0.71-1.12	0.90	0.72-1.14
**All-cause death**	1.65	1.15-2.38	0.81	0.63-1.04	0.81	0.63-1.04
**Cardiovascular death**	1.63	0.87-3.04	1.12	0.73-1.72	1.12	0.73-1.73
**Any TE**	3.77	1.36-10.45	1.27	0.79-2.05	1.34	0.83-2.15
**Major bleeding**	1.49	0.69-3.19	0.88	0.52-1.50	0.86	0.50-1.47

*Adjusted for age.

Composite outcome of all-cause death/any thromboembolism

TE – thromboembolism; CI – confidence interval.

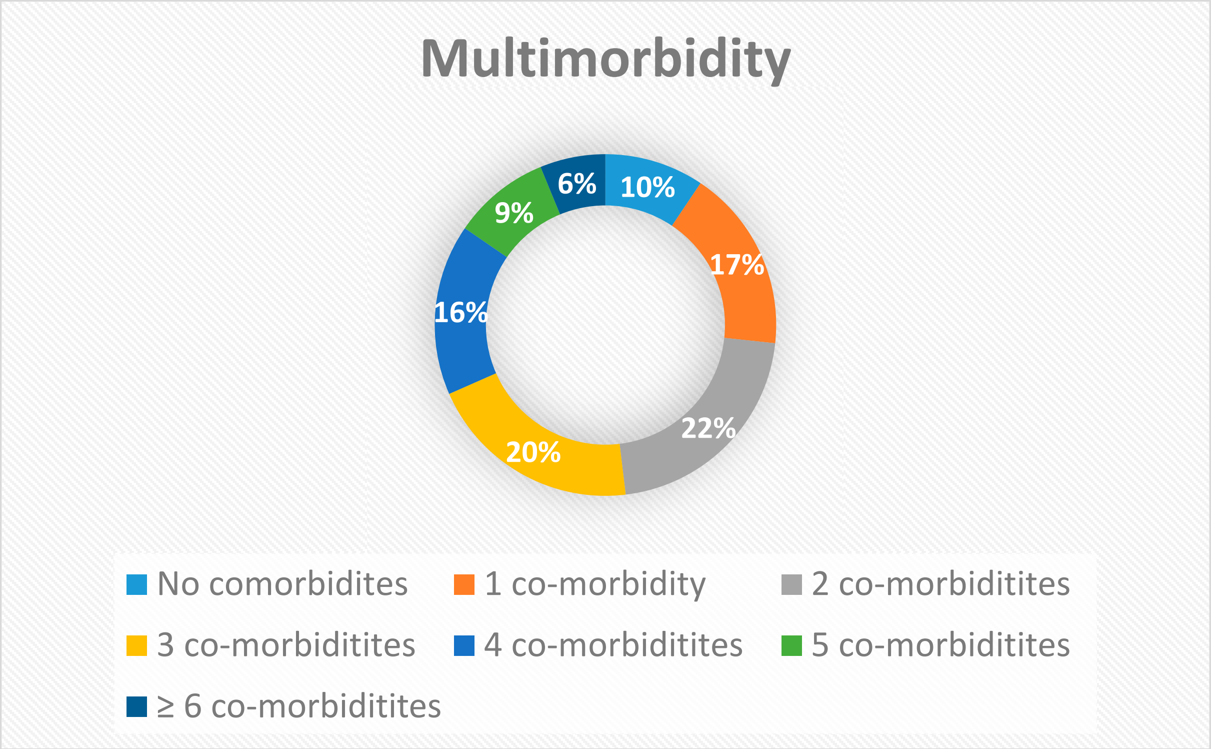

Data Supplement Figure S1

Proportion of patients with atrial fibrillation according to number of comorbidities.

ChiOTEAF Registry Investigators

Academic Executive Steering Committee

Gregory Y H Lip, MD, Liverpool Centre for Cardiovascular Science, University of Liverpool and Liverpool Heart & Chest Hospital, Liverpool, United Kingdom (Co-Chair)

Xiaoying Li, MD, PhD, Department of Geriatric Cardiology, Chinese PLA General Hospital, Beijing, China (Co-Chair)

Yutang Wang, MD, PhD, Department of Geriatric Cardiology, Chinese PLA General Hospital, Beijing, China (Co-Chair)

Changsheng Ma, MD, PhD, Department of Cardiology, Center for Atrial Fibrillation, Beijing Anzhen Hospital, Capital Medical University, Beijing, China

Shu Zhang, MD, PHD, Fuwai Hospital, Chinese Academy of Medical Sciences, Beijing, China

Congxin Huang, MD, PHD, RenMin Hospital, Wuhan University, Wuhan, China

Jiefu Yang, MD, PhD, Department of Cardiology, Beijing Hospital, Chinese Academy of Medical Sciences and Peking Union Medical College, Beijing, China.

Meilin Liu, Department of Geriatrics, Peking University First Hospital, Beijing, China

Data Management Committee

Gregory Y H Lip, MD, Liverpool Centre for Cardiovascular Science, University of Liverpool and Liverpool Heart & Chest Hospital, Liverpool, United Kingdom

Yutao Guo, MD, PhD, Department of Pulmonary Vessel and Thrombotic Disease, Sixth Medical Centre, Chinese PLA General Hospital, Beijing, China

Guangliang Shan, PhD, Department of Epidemiology and Statistics, Institute of Basic Medical Sciences, Chinese Academy of Medical Sciences and School of Basic Medicine, Peking Union Medical College, Beijing, China

Taixiang Wu, MD, PhD, Administrator of Chinese Clinical Trial Registry, Associate Professor of Clinical Epidemiology and Evidence-Based Medicine, West China Hospital, Sichuan University

Chen Yao, PhD, Associate Director, Peking University Clinical Research Institute, Beijing, China

Steering Committee Members

Changsheng Ma, MD, PhD	Anzhen Hospital, Capital Medical University, Beijing
Congchun Huang, MD, PhD	Air Force General Hospital, Beijing
Cuntai Zhang, MD, PhD	Tongji Hospital, Tongji Medical college, Huazhong University of Science & Technology, Guangzhou
Dang Aiming, MD, PhD	Fuwai Hospital, Chinese Academy of Medical Sciences, Beijing
Dawei Qian, MD, PhD	Ji Lin Hospital, Ji Lin
Fakuan Tang, MD, PhD	PLA 309th Hospital, Beijing

Fang Wu, MD, PhD	Rui Jin Hospital, Tong University School of Medicine, Shanghai
Feng Liu, MD, PhD	First People's Hospital, Guangdong
Gexin Zhu, MD, PhD	The General Hospital, Tianjing Medical Hospital, Tianjing
Guo Yutao, MD, PhD	PLA General Hospital, Beijing
Guorong Xi, MD	Health Division of Guard Bureau, Chinese PLA General Staff Department, Beijing
Heng Dou, MD, PhD	Beijing Hospital, Beijing
Hou Cuihong, MD, PhD	Fuwai Hospital, Chinese Academy of Medical Sciences, Beijing
Hua Li, MD, PhD	The First Affiliated Hospital, Zhengzhou University, Zhejiang
Hui Han, MD, PhD	The First Affiliated Hospital, Harbin Medical University, Heilongjiang
Huiliang Liu, MD, PhD	Wujing General Hospital, Beijing
Jian Kong, MD, PhD	The First Affiliated Hospital, Ji Lin University, Ji Lin
Junxia Li, MD, PhD	Beijing PLA General Hospital, Beijing
Liang Zaoguang	The First Affiliated Hospital, Harbin Medical University, Heilongjiang
Liangyi Si, MD, PhD	Southwest Hospital, Chongqing
Liu Meilin, MD, PhD	The First Affiliated Hospital, Peking University First Hospital, Beijing

Liu Yanxia, MD	Shenyang General PLA Hospital
Liu Yu, MD	Yanggu People's Hospital, Shandong
Liu Zhiming, MD, PhD	Fuwai Hospital, Chinese Academy of Medical Sciences, Beijing
Luo Ma, MD, PhD	NAVY General Hospital, Beijing
Ming Li, MD, PhD	Beijing Friendship Hospital, Capital Medical University, Beijing
Qian Xiao, MD, PhD	First Affiliated Hospital, Chongqing Medical University, Chongqing
Qingwei Chen, MD, PhD	The Second Affiliated Hospital, Chongqing Medical University, Chongqing
Qiong Chen, MD, PhD	Xiangya Hospital, Central South University, Hunan
Ren Xuejun, MD, PhD	Anzhen Hospital, Capital Medical University, Beijing
Shan Zhaoliang, MD, PhD	PLA General Hospital, Beijing
Shi Xiangming, MD, PhD	PLA General Hospital, Beijing
Shilian Hu, MD, PhD	Anhui Provincial Hospital, Anhui
Song Bai, MD, PhD	First Affiliated Hospital of Kunming Medical University, Kunming
Tianchang Li, MD, PhD	NAVY General Hospital, Beijing
Wang Lijuan, MD	Suqian People's hospital, Jiangsu

Wu Qiang, MD, PhD	Guizhou Provincial People's Hospital
Xianghu Wang, MD, PhD	Union Hospital, Tongji Medical College, Huazhong University of Science & Technology, Guangzhou
Xiaojuan Bai, MD, PhD	Sheng Jing Hospital, China Medical University, Shengyang, Liaoning
Xiaoming Wang, MD, PhD	Xijing Hospital, Xian
Xinchun Yang, MD, PhD	Chao-Yang Hospital, Capital Medical University, Beijing
Xuan He, MD, PhD	Air Force General Hospital, Beijing
Xuejun Liu, MD, PhD	The First Affiliated Hospital, Shanxi Medical University, Shanxi
Yan Li, MD, PhD	First People's Hospital, Kunming, Yunnan
Yang Jiefu, MD, PhD	Beijing Hospital, Beijing
Yong Wang, MD, PhD	China-Japan Friendship Hospital, Beijing
Yunmei Yang, MD, PhD	The First Affiliated Hospital, Zhenjiang University, Zhejiang
Zeng Yuan, MD, PhD	PLA 306 Hospital
Zhang Shu, MD, PhD	Fuwai Hospital, Chinese Academy of Medical Sciences, Beijing
Zhang Wei, MD, PhD	Beijing PLA General Hospital, Beijing
Zhanyi Lin, MD, PhD	Guangdong General Hospital, Guangdong
